# Emerging role of mesenchymal stem cells-derived extracellular vesicles in vascular dementia

**DOI:** 10.3389/fnagi.2024.1329357

**Published:** 2024-02-08

**Authors:** Ziying Liu, Lin Cheng, Lushun Zhang, Chunxiao Shen, Shufei Wei, Liangliang Wang, Yuemin Qiu, Chuan Li, Yinyi Xiong, Xiaorong Zhang

**Affiliations:** ^1^Department of Pathology, Affiliated Hospital of Jiujiang University, Jiujiang, Jiangxi, China; ^2^Jiujiang Clinical Precision Medicine Research Center, Jiujiang, Jiangxi, China; ^3^Department of Neurology, Affiliated Hospital of Jiujiang University, Jiujiang, Jiangxi, China; ^4^Department of Rehabilitation, Affiliated Hospital of Jiujiang University, Jiujiang, Jiangxi, China; ^5^Center for Cognitive Science and Transdisciplinary Studies, Jiujiang University, Jiujiang, Jiangxi, China

**Keywords:** vascular dementia, extracellular vesicles derived from mesenchymal stem cells, pathophysiology, neuroinflammation, diagnosis and treatment

## Abstract

Vascular dementia (VD) is a prevalent cognitive disorder among the elderly. Its pathological mechanism encompasses neuronal damage, synaptic dysfunction, vascular abnormalities, neuroinflammation, and oxidative stress, among others. In recent years, extracellular vesicles (EVs) derived from mesenchymal stem cells (MSCs) have garnered significant attention as an emerging therapeutic strategy. Current research indicates that MSC-derived extracellular vesicles (MSC-EVs) play a pivotal role in both the diagnosis and treatment of VD. Thus, this article delves into the recent advancements of MSC-EVs in VD, discussing the mechanisms by which EVs influence the pathophysiological processes of VD. These mechanisms form the theoretical foundation for their neuroprotective effect in VD treatment. Additionally, the article highlights the potential applications of EVs in VD diagnosis. In conclusion, MSC-EVs present a promising innovative treatment strategy for VD. With rigorous research and ongoing innovation, this concept can transition into practical clinical treatment, providing more effective options for VD patients.

## Graphical abstract

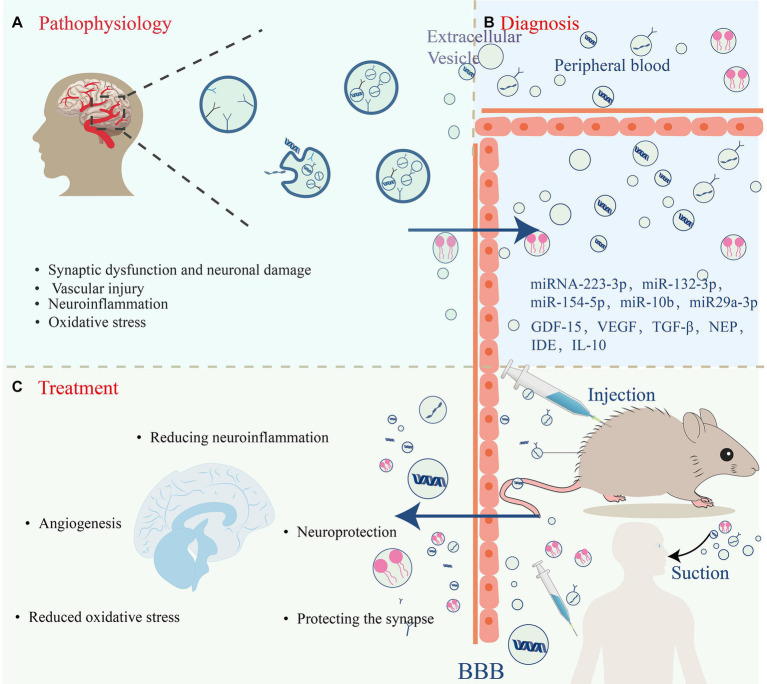

## 1 Introduction

Dementia is characterized by an acquired progressive cognitive decline in memory and other cognitive domains, that are severe enough to interfere with daily living or occupational functioning (Livingston et al., 2020). Vascular dementia (VD) ranks second only to Alzheimer’s disease (AD) as a cause of dementia, representing approximately 15% of total cases of dementia (O’Brien and Thomas, 2015). Other terms for VD include vascular cognitive impairment dementia and multi-infarct dementia (Akhter et al., 2021). In 2019, an estimated 55.2 million people globally were afflicted with dementia. Projections suggest that by 2030, the number of dementia patients will increase to approximately 78 million, with the global cost associated with dementia surging to 1.7 trillion US dollars (WHO, 2021). As the global population ages, the incidence of dementia is increasing significantly, predicting a twofold increase in AD and other related dementias by 2050 (Guest and Smith, 2021). Currently, the effective treatment options of VD are still limited (O’Brien and Thomas, 2015). As a prevalent neurodegenerative disease in older individuals (Hase et al., 2020), VD’s association with vascular, neuronal, and synaptic dysfunctions complicates its treatment. With a rapidly aging global population, there is an escalating urgency for VD treatment solutions. Thus, researchers are compelled to investigate innovative diagnostic and therapeutic strategies to address this challenge.

Mesenchymal stem cells (MSCs) have become a focal point in medical research. Nonetheless, their clinical application faces hurdles like storage issues, reduced cell viability post-transplantation, inefficient targeting, dose determination to sustain therapeutic effects, and cellular aging due to *in vitro* expansion (Regmi et al., 2019; Al-Khawaga and Abdelalim, 2020; Yen et al., 2020; Yu et al., 2020). An increasing body of research suggests that MSCs do not solely operate through cell differentiation; they also mediate myriad biological effects through their secreted extracellular vesicles (MSC-EVs) (Asgarpour et al., 2020). Moreover, MSC-EVs offer several advantages over MSCs, including enhanced targeted delivery, reduced immunogenicity, and superior reparative potential. Consequently, MSC-EVs could introduce novel therapeutic pathways, ushering in exciting prospects for clinical application (Tang et al., 2021).

The primary aim of this paper is to unearth the potential applications of MSC-EVs in VD diagnosis and treatment. By systematically analyzing the bioactive components within EVs and their roles in neuroprotection, anti-inflammation, and antioxidation, we intend to underscore their viability in VD therapy. This study will also emphasize the prospective role of EVs in the early detection of VD, particularly their potential as biomarkers. Additionally, we will probe current challenges and forthcoming research avenues, steering the precise and efficient use of MSC-EVs in advancing VD treatments. In essence, through a thorough exploration of MSC-EVs’ role in VD treatment, we aim to lay the groundwork to tackle this clinical challenge, offering enhanced therapeutic alternatives for patients and fostering the health and wellness of an aging population.

## 2 Overview of MSCs and EVs

**Figure 1:**
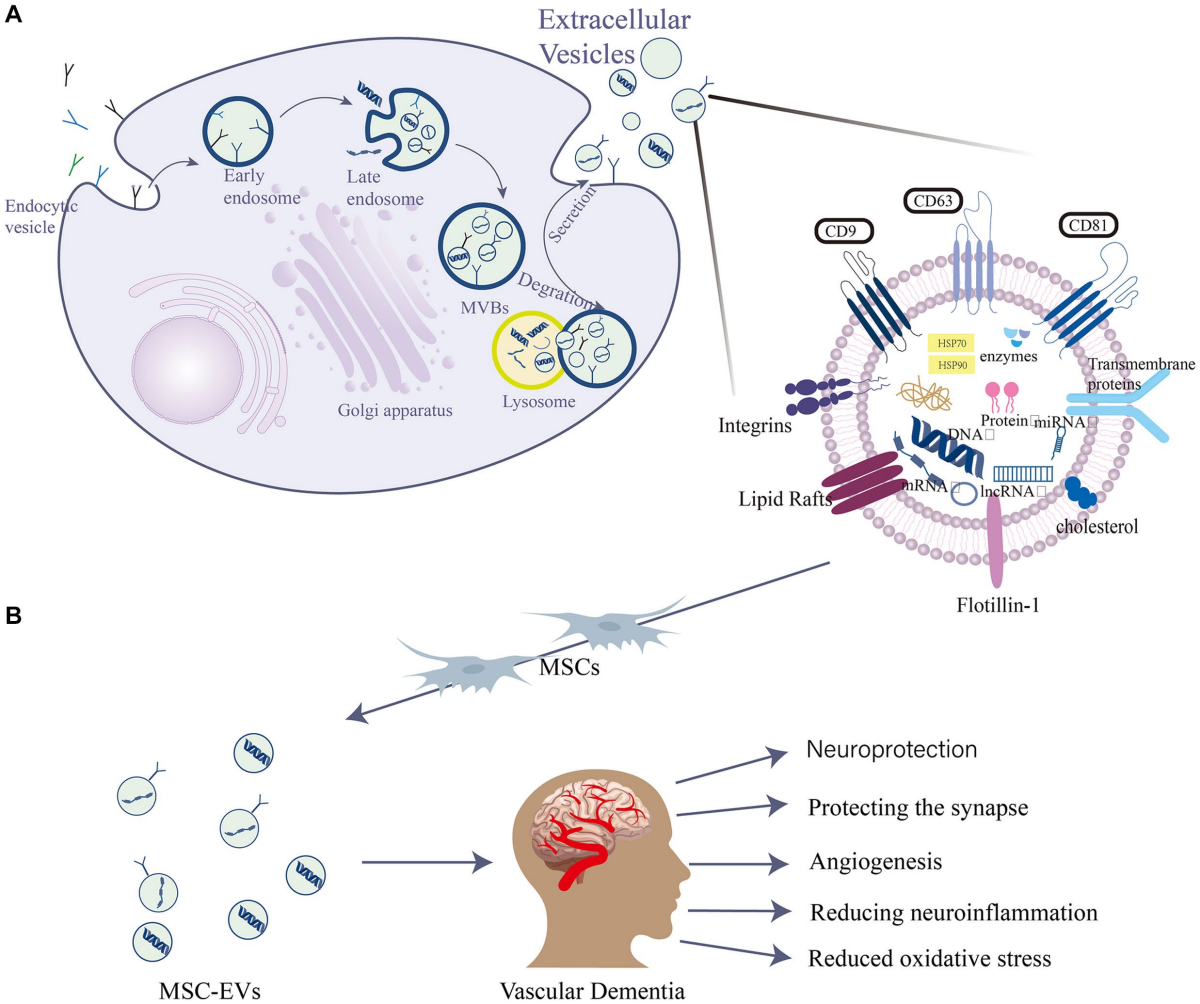
EVs are intraluminal vesicles formed by budding inward from the endosomal membrane during the maturation of MVBs, which are intermediates in the endosomal system. MVBs can be fused to autophagosomes or lysosomes for degradation. Exocytosis results in the release of EVs. **(A)** EVs contain different forms of cell surface proteins, signaling proteins, nucleic acids, amino acids, metabolites, and proteins. **(B)** Role of mesenchymal stem cell (MSC)-EVs in vascular dementia (VD): MSC-EVs have neuroprotective, angiogenic, and synapse-protective effects and can also reduce the progression of VD by reducing neuroinflammation and oxidative stress.

EVs are widely studied in biomedical applications due to their biocompatibility, appropriate size, and low immunogenicity, which collectively contribute to an extended circulation time (Peng and Mu, 2016; Yue et al., 2023). The general term “EVs” contain various types of membrane-enclosed vesicles, including exosomes, extracellular autophagic vesicles, and apoptotic bodies, and these can have overlapping size ranges (Davidson et al., 2023). Their diameters are 30–150 nm, 200–1,000 nm and 800–5,000 nm, respectively (He et al., 2018). However, there is currently no precise method to distinguish and isolate exosomes from other EVs (Théry et al., 2018). Many studies have convincingly shown that both exosomes and microvesicles contain specific proteins and nucleic acids that act as vectors for intercellular communication factors (Davidson et al., 2023; Du et al., 2023). They have since been established as being secreted by neural progenitor cells (Marzesco et al., 2005), MSCs (Lai et al., 2010), CD34^+^ stem cells (Sahoo et al., 2011), and other progenitor cell types. EVs are intraluminal vesicles formed by the inward budding of the endosomal membrane during the maturation of multivesicular bodies (MVBs). They act as intermediaries within the endosomal system and are released when MVBs fuse with the cell surface (van Niel et al., 2018). They are also characterized by specific marker proteins such as the tetraspanins CD63, CD81, and CD9 (McAndrews and Kalluri, 2019; Figure 1). They facilitate the transportation of various biochemical substances, including cytokines, mRNA, miRNA, and protein (Wang et al., 2023). Serving as mediators for cell-to-cell communication, they transport proteins, lipids, nucleic acids, and other components to neighboring or distant cells (Li S. P. et al., 2018). The protein content of EVs has been thoroughly identified through various proteomic methods (Welton et al., 2010). Mass spectrometry reveals over 4,000 distinct proteins present in EVs (Tickner et al., 2014). EVs also exhibit high stability in various body fluids, including blood, urine, pleural effusion, peritoneal effusion, cell supernatant, milk, saliva, and cerebrospinal fluid (CSF) (Skog et al., 2008). Over the past two decades, the scientific interest in EVs has surged, with annual citations leaping from 28 in 1996 to 24,765 in 2016, marking EVs as a current research hotspot (Marban, 2018).

MSCs are pluripotent stem cells known for their self-renewal and multi-directional differentiation capabilities. Initially isolated from bone marrow (Schulman et al., 2018), they can differentiate into various cell types under different stimuli (Narakornsak et al., 2016). Due to their versatile differentiation potential and self-renewal ability, MSCs hold promising research and application prospects in tissue engineering, regenerative medicine, and immunotherapy (Merimi et al., 2020). As a therapeutic modality, MSCs have been successfully applied to numerous diseases (Margiana et al., 2022). Moreover, MSCs orchestrate immune responses by secreting immunomodulatory factors paracrinally, fostering new blood vessel formation, supplying nutrients to damaged neurons, and promoting nerve tissue repair and regeneration (Harrell et al., 2021). Given their extensive functions in nerve cells, including self-renewal, anti-inflammatory action, signal transduction, differentiation (Torres Crigna et al., 2018), potent immunosuppressive, vasoregulatory properties, and no ethical controversies (Salgado et al., 2015), MSCs have potential applications in a variety of treatments related to neurodegenerative diseases (Torres Crigna et al., 2018). As a result, they have emerged as a focal point in neurodegenerative diseases (Shariati et al., 2020). More than 2,000 patients at different stages of neurodegenerative diseases have received MSC treatments, with most reporting positive outcomes (Ghasemi et al., 2023). However, the exact paracrine action mechanism of MSCs remains elusive. Recent studies suggest that in addition to secreting various soluble factors, MSCs release a significant number of EVs. These EVs play a pivotal role in intercellular communication, influencing not just normal physiological processes but also the development and progression of diseases (Rani et al., 2015).

As alluded to earlier, many of the immunomodulatory effects attributed to MSCs are due to the properties of MSC-EVs (Rani et al., 2015). These EVs are released from the plasma membrane into the extracellular environment, exerting biological effects through paracrine and endocrine mechanisms (Harrell et al., 2019a). For instance, miRNA concentrations in EVs surpass those in parental cells and body fluids (Sun et al., 2018). Once MSC-EVs enters the receptor cells, miRNA is released to further target and silence the mRNA of related proteins, thus affecting the physiological function of the receptor cells (Wang et al., 2022). miRNA, especially miR-146 and miR-21, can modulate the phenotype, function, and activity of nerve and immune cells (Harrell et al., 2019a) and miR-132-3p can change synaptic dysfunction (Ma et al., 2022). Therefore, it is considered to be a crucial factor in the beneficial role of MSC-EVs in the treatment of neuroinflammatory diseases and neurodegenerative diseases. The therapeutic potential of MSC-EVs has been explored in a plethora of neurodegenerative disease models, including AD, VD, multiple sclerosis, stroke, neuroinflammation, traumatic brain injury, spinal cord injury, and status epilepticus (Guy and Offen, 2020). Furthermore, employing MSC-EVs can sidestep the adverse reactions triggered by the exogenous administration of MSCs (Guy and Offen, 2020). Hence, MSC-EVs are viewed as a promising alternative to MSCs in treating inflammatory and degenerative neurological disorders.

## 3 Role and application of MSC-EVs in the pathophysiological process of VD

Mangy underlying pathophysiological processes that lead to vascular brain injury in VD, including hypoperfusion, endothelial dysfunction, blood–brain barrier breakdown, synaptic dysfunction, inflammation, oxidative stress, hypoxia, and nerve cell injury (Hosoki et al., 2023). VD is a multifaceted neurological disorder, with its progression involving various cell types and molecular processes. These mechanisms are interconnected and together drive the onset and progression of VD (O’Brien and Thomas, 2015). EVs play roles in intercellular communication and in the diagnosis and treatment of VD. Consequently, the investigation of MSC-EVs is of significant interest. MSC-EVs not only contain abundant bioactive molecules but also function as vital mediums for information transfer between cells (Joo et al., 2023). They are believed to have a central role in VD diagnosis and treatment; for instance, MSC-EVs can deliver neuroprotective factors that encourage neuronal survival and repair, thus reducing neuronal damage and VD severity (Ma et al., 2022). Additionally, they can enhance regular communication between neurons by modulating synaptic plasticity. Moreover, MSC-EVs possess anti-inflammatory and antioxidative properties, potentially mitigating neuroinflammation and oxidative damage in VD (Harrell et al., 2021). Overall, MSC-EVs have roles in VD pathophysiology and may aid in the development of targeted treatments and neuroprotective strategies. We will delve into the pathophysiological roles of MSC-EVs in VD in the subsequent sections of this study.

### 3.1 Role of MSC-EVs in synaptic dysfunction and neuronal damage in VD

Similar to cognitive disorders such as AD, neuronal injury, and synaptic dysfunction are primary drivers of VD (Iadecola, 2013). Brain information processing demands a continuous, high energy supply (Faria-Pereira and Morais, 2022), with the majority being used to restore ion movement essential for neuron communication and neurotransmitter uptake. Cerebral ischemia in VD patients leads to neuronal hypoxia and insufficient energy supply, resulting in neuronal damage (Lopez-Domenech and Kittler, 2023), while synaptic dysfunction hampers the nervous system’s communication (Lepeta et al., 2016), affecting perception, movement, learning, and cognitive functions. Therefore, preserving neuronal density and promoting synaptic formation and plasticity are vital to improve cognitive deficits post-VD (Ma et al., 2022).

Recently, some experimental and clinical studies demonstrated that MSC-EVs were found to possess tissue repair and regenerative functions akin to MSCs (Yang Z et al., 2022). MSC-EVs can stimulate the axonal growth of neurons (Zhang et al., 2017). The utilization of EVs extracted from bone marrow mesenchymal stem cells of type 2 diabetes mellitus rats (T2DM-MSCs-Exos) significantly improves axonal density and myelin phospholipid density (Venkat et al., 2020). Overexpression of specific miRNAs, such as miR-23a (Du et al., 2020), miR-200 (Fu et al., 2019), miR-133b (Li D et al., 2018), miR-17-92 (Yang et al., 2017), and miR-132-3p (Ma et al., 2022), has been shown to enhance neural gene expression and myelination. Within this context, EVs transfer these miRNAs from the neuronal cell body to the axon, thereby facilitating axon outgrowth (Liu et al., 2023). For instance, Yang et al. highlighted the miR-17-92 cluster’s role in neuronal and vascular plasticity, presenting it as a potential therapeutic strategy for injury to the central nervous system (Yang et al., 2017). Ma et al. (2022) demonstrated that miR-132-3p could improve synaptic and cognitive functions, while also mitigating neuronal damage in VD mice, through the activation of the Ras/Akt/GSK-3β signaling pathway.

Additionally, MSC-EVs have roles in nerve injury repair. One study evaluated the impact of EVs derived from adipose-derived MSCs (adMSCs) on neuronal growth and discovered nerve growth factor mRNA transcripts such as BDNF, FGF-1, GDNF, IGF-1 and NGF, in adMSCs-derived extracellular vesicles for the first time. These findings suggest a potential therapeutic application of these EVs for tissue-engineered nerves (Bucan et al., 2019). EVs-contained growth differentiation factor-15 (GDF-15) has neuroprotective effects via the AKT/GSK-3β/β-catenin pathway (Xiong et al., 2021). Chen et al. found that EVs directly sourced from MSCs could inhibit astrocyte activation, recover the expression of genes associated with neuronal memory and synaptic plasticity, and enhance cognitive function (Chen et al., 2021). *In vitro* experiments, MSCs-derived exosomal miR-455-3p targeted PDCD7 to alleviate neuronal injury and injury of N2a cells (Gan and Ouyang, 2022). Such effects were also observed in non-human primate models. Here, MSC-EVs, delivered with EVs derived from MSCs, reduced physiological and morphological changes related to neuronal injury around lesions (Medalla et al., 2020). Exosome biogenesis mechanisms might also possess neuroprotective properties, with MSC-EVs potentially aiding in the clearance of misfolded proteins, thereby exerting detoxification and neuroprotection (Kalluri and LeBleu, 2020). *In vitro* experiments have shown that MSC-EVs promoted neural progenitor cell proliferation after stroke (Zhou et al., 2020).

In the context of MSC-EVs’ role in synaptic dysfunction and neuronal damage in VD, while the exact mechanisms warrant further exploration, current insights offer promising therapeutic avenues. These findings form a robust foundation for subsequent research, with the potential to revolutionize treatments for neurodegenerative diseases such as VD.

### 3.2 MSC-EVs and vascular injury in VD

**Figure 2:**
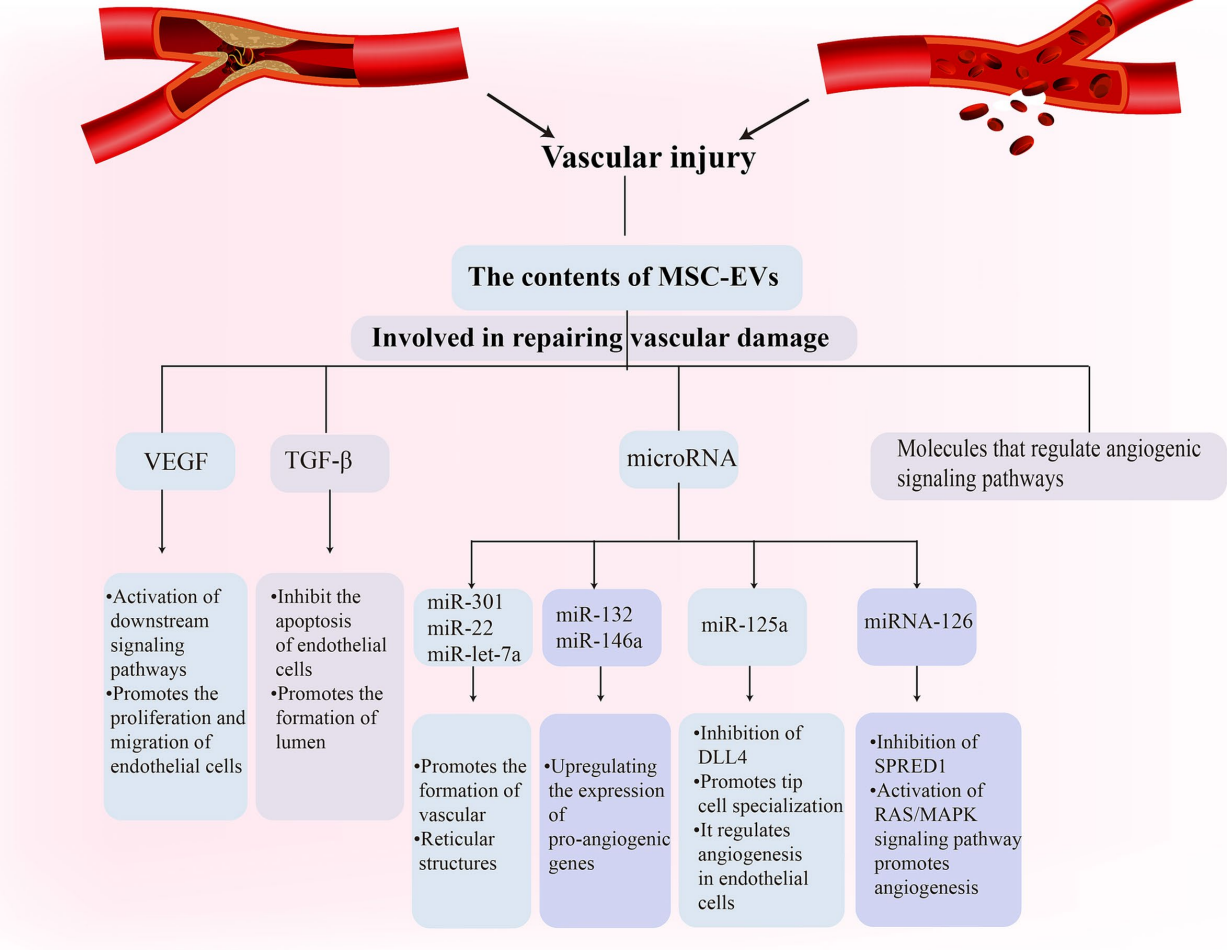
Mesenchymal stem cell (MSC)-Exos participate in the vascular repair process in vascular dementia (VD) through a variety of ways.

Vascular damage is a fundamental pathological aspect of VD (Kalaria, 2018; Chang Wong and Chang Chui, 2022). MSC-EVs have been recognized for establishing specific microenvironments that regulate vessel density and function (Watt et al., 2013; Bronckaers et al., 2014). They foster the healing of ischemic tissue-linked disorders through proteins that induce angiogenesis (Anderson et al., 2016). Notably, MSC-EVs contain potent angiogenic paracrine effectors (Anderson et al., 2016). These molecules can influence endothelial cell behavior either through receptor binding or intracellular signaling pathway modulation. For instance, EVs vascular endothelial growth factor (VEGF) can attach to endothelial cell receptors and activate subsequent signaling pathways, facilitating endothelial cell proliferation and migration (Olejarz et al., 2020). Additionally, EVs transforming growth factor-β (TGF-β) can prevent endothelial cell apoptosis and promote tube formation. Research has shown that MSC-EVs derived from bone marrow can support angiogenesis, inhibit IFN-γ secretion by peripheral blood monocytes, and contain immune-related microRNAs [miR-let-7a (Cho et al., 2015), miR-301 (Li et al., 2019), miR-22 (Wang X et al., 2020), etc.]. They can foster the development of blood vessels and network structures in human umbilical vein endothelial cells (Heo and Kim, 2022). Furthermore, specific exosome-released microRNAs (miR-132 and miR-146a) upregulate pro-angiogenic gene expression and enhance human umbilical vein endothelial cell growth and tube formation. adMSC-EVs also play a critical role in angiogenesis, with miR-125a in adMSCs being transferred to endothelial cells through EVs, thereby regulating endothelial cell angiogenesis through direct inhibition of its target, delta-like 4 (DLL4), which, in turn, fosters tip cell specialization (Liang et al., 2016). MSC-EVs can also bolster neovascularization by influencing angiogenesis-related signaling pathways. microRNA in EVs can regulate the activity of angiogenesis-related signaling pathways by targeting the mRNA of specific genes. For instance, EVs miRNA-126 can target and downregulate the SPRED1 gene, activating the RAS/MAPK signaling pathway to promote angiogenesis (Fish et al., 2008; Rom et al., 2021; Figure 2).

It is crucial to note that this field remains under active investigation. More clinical studies are essential to ascertain the safety and efficacy of MSC-EVs in treating vascular damage in VD. Nonetheless, current studies hint at the promising potential of MSC-EVs in addressing vascular damage related to VD.

### 3.3 MSC-EVs and neuroinflammation in VD

Several studies have demonstrated that neuroinflammation is a pivotal process in the progression of most neurodegenerative diseases (Tejera et al., 2019). Neuroinflammation refers to an immune response mediated by glial cells in the central nervous system, the primary location of innate immunity (Tian et al., 2022). This inflammatory response plays a significant role in the etiology of VD (Belkhelfa et al., 2018), contributing to its onset and development. Evidence exists of an inflammatory reaction in the brains of VD patients, with inflammatory factors released by activated microglia and astrocytes (Yang Y et al., 2022) potentially leading to neuronal damage and cognitive dysfunction. Furthermore, neuroinflammation might also promote the deposition of Aβ amyloid and subsequent neuronal death (Ising et al., 2019), thereby worsening the severity of VD.

Microglia, the primary immune response cells in the inflammatory response of the central nervous system (Shields et al., 2020), play a dual role in regulating the inflammatory response during pathophysiological changes (Yang Y et al., 2022). These activated microglia can exhibit two phenotypes: a classically activated proinflammatory M1 and an alternatively activated anti-inflammatory M2 phenotype (Orihuela et al., 2016). M1 microglia release nitric oxide (NO) and proinflammatory cytokines [tumor necrosis factor [TNF]-α, interleukin [IL]-1β, and IL-15 (Gao and Hong, 2008)], intensifying neuroinflammation, hindering synaptogenesis, compromising cognitive function (Wang et al., 2018), and facilitating VD progression. Conversely, M2 cells produce Aβ-degrading enzymes (NEP), insulin-degrading enzymes (IDE), and anti-inflammatory cytokines (IL-10 and TGF-β), which reduce Aβ deposition, diminish inflammation (Subhramanyam et al., 2019), and shield neurons from damage. Ding and colleagues discovered that MSC-EVs can modulate microglial activity and decrease their inflammatory reactions, guiding microglial polarization toward an immunosuppressive M2 phenotype (Subhramanyam et al., 2019). Specifically, miRNAs such as miR-216a-5p (Liu et al., 2020), miR-125a (Chang et al., 2021) and miR-146a-5p (Zhang et al., 2021) present in MSC-EVs can reduce the levels of inflammatory factors and pro-inflammatory microglia after acute injury in the central nervous system and can promote the conversion of the M1 phenotype to the M2 phenotype. Furthermore, treatment with T2DM-MSC-EVs significantly reduces the expression of activated microglia, M1 macrophages, as well as the inflammatory factors MMP-9 and MCP-1. The MiR-9/ABCA1 pathway may play a crucial role in the attenuation of inflammation induced by T2DM-MSC-EVs (Venkat et al., 2020). These miRNAs contribute to anti-inflammatory property and nerve injury recovery effect. The miRNA carried by MSC-EVs act through specific pathways; for instance, MSC-EVs suppress NF-κB phosphorylation by targeting TNF receptor-associated factor 6 (TRAF6) and IL-1 receptor-associated kinase 1 (IRAK1) in microglia through miR-146a-5p (Zhang et al., 2021). Being a pivotal inducible transcription factor in microglia, NF-κB significantly influences immune and inflammatory regulation within the central nervous system. Most anti-inflammatory medications operate by inhibiting this pathway (Kaltschmidt et al., 2005). Inhibition of NF-κB signaling curtails the gene expression of inducible NO synthase, TNF-α, IL-1β, and IL-6, thereby preventing the formation of the M1 phenotype in MSC-EVs-treated microglia (Nakano et al., 2020). In MSC-EVs-treated a-βpp/PS1 mice, levels of NEP, IDE, IL-10, and TGF-β were notably elevated, whereas inflammatory factors (TNF-α and IL-1β) were significantly reduced. *In vitro* experiments also confirmed this alternative microglia activation by MSC-EVs (Ding et al., 2018).

**Figure 3:**
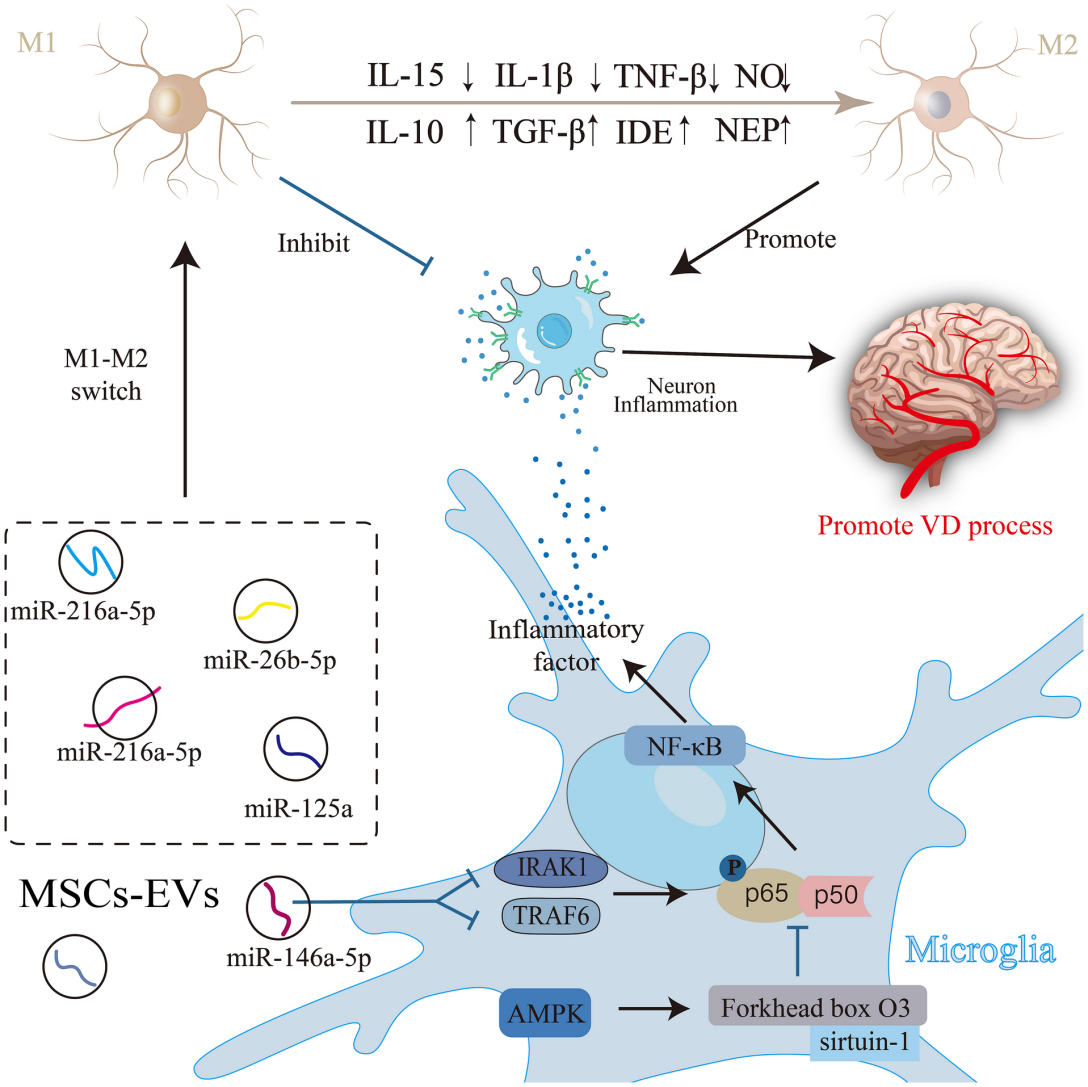
Mesenchymal stem cell (MSC)-Exos are involved in the process of neuroinflammation in vascular dementia (VD). Activated microglia have two phenotypes: the classically activated pro-inflammatory M1 phenotype and the alternatively activated anti-inflammatory M2 phenotype. Substances in MSC-EVs can regulate the activity of microglia through related pathways, induce the polarization of microglia to the immunosuppressive M2 phenotype, or directly inhibit the release of inflammatory factors to alleviate neuroinflammation, and improve VD.

Adenosine monophosphate-activated protein kinase (AMPK) is among the foremost endogenous neuroprotective agents against inflammatory reactions. Its activation results in the increased phosphorylation of endothelial NO synthase, promoting nerve regeneration (García-Prieto et al., 2015). Activation of the AMPK signaling pathway also promotes microglial polarization toward the M2 phenotype. Additionally, it can modulate NF-κB activity through downstream proteins, such as sirtuin-1 and Forkhead box O3, thereby restricting the release of inflammatory agents and exerting an anti-inflammatory effect (Wang et al., 2013). This reduces the neuroinflammatory response. Evidence suggests that MSC-EVs provide neuroprotection by suppressing NF-κB and stimulating the AMPK signaling pathway to alleviate neuroinflammation (Han et al., 2021; Figure 3).

Several studies have indicated that CD4 + T cells infiltrate the central nervous system during neurodegeneration (Korn and Kallies, 2017). These activated CD4 + T cells can readily penetrate the blood–brain barrier (BBB) (Engelhardt and Ransohoff, 2005). Upon reaching the injury site, these cells perform various functions based on their phenotype (Luckheeram et al., 2012). Their actions may be linked to the elimination of intracellular pathogens, consequently resulting in neuroinflammation and neuronal damage within the central nervous system (Solleiro-Villavicencio and Rivas-Arancibia, 2018). MSC-EVs can mitigate this neuroinflammation by suppressing the activity of infiltrating CD4 + T cells at the site of brain inflammation *in vivo* (Alvarez et al., 2018). In conclusion, MSC-EVs may play a role in the pathophysiology of VD by influencing the evolution of neuroinflammation. Nonetheless, these connections require further exploration in research. Grasping these interconnections can pave the way for more effective treatments.

### 3.4 The role of MSC-EVs in the oxidative stress of VD

Oxidative stress damage is characterized by a situation where oxygen and its derivative free radicals surpass a cell’s inherent antioxidant defense capacity (Bisht et al., 2017). Reactive oxygen species (ROS) release-induced oxidative stress is pivotal in neuronal death and the onset of neurodegenerative diseases (Tournier et al., 2023). Cerebrovascular diseases (CSVD) lead to cerebral ischemia, hypoxia, vascular endothelial injury, and ensuing inflammatory responses, which give rise to oxygen free radicals, culminating in oxidative stress (Chukanova et al., 2022). Concurrently, atherosclerosis stands as a key risk factor and pathological hallmark of VD (Wolters and Ikram, 2019), with oxidative stress central to the key mechanism of pathological changes of atherosclerosis (Kattoor et al., 2017). Hence, oxidative stress is postulated as a chief factor in VD’s foundational mechanisms, and augmented damage is suggested as a central process behind cognitive lapses in VD (Iadecola, 2013).

MSC-EVs exhibit unique advantages in suppressing oxidative damage through their antioxidative activity (Zhang W et al., 2023). MSC-EVs and EVs -derived agents possess potent antioxidant capabilities, capable of neutralizing excess ROS (Xia et al., 2021) within cells through antioxidant enzymes and molecules, including glutathione peroxidase (GPX). This can alleviate oxidative stress or boost calcium influx, thus curtailing proinflammatory factor concentrations and dampening ROS production (Wang T et al., 2020). Research indicates that EVs contain a series of upregulated antioxidant miRNAs, such as miR-215-5p, miR-424-5p, miR-31-3p, miR-193b-3p, and miR-200b-3p. This suggests a crucial role of EVs miRNAs in antioxidative stress (Luo et al., 2021). Liu et al. discerned an upsurge in Nrf2 and HO-1 expressions post MSC-EVs treatment, consequently augmenting the cells’ antioxidant potential and ameliorating cognitive deficits in mice (Liu et al., 2022). Moreover, as previously noted, MSC-EVs can temper neuroinflammation—a prevalent instigator of oxidative stress—potentially lessening oxidative stress intensity through inflammation reduction. For instance, they can exert their effects through various pathways, including the NF-kB signaling pathway (Yan et al., 2022) and the Nrf2/Keap1 signaling pathway (Tang et al., 2023). Additionally, they have the ability to modulate mitochondrial membrane potential and mitigate mitochondrial ROS production (Xian et al., 2019). Zhang et al. demonstrated through experiments that MSC-Exo treatment significantly ameliorated the elevated levels of oxidative stress (Zhang X et al., 2023). In addition, MSC-EVs can ameliorate oxidative stress by reducing ROS generation, mitigating DNA damage, normalizing calcium signaling, and modulating mitochondrial alterations. Furthermore, they enhance antioxidant capacity (Wang T et al., 2020). Numerous studies suggest that EVs derived from MSCs could be protective in modulating oxidative stress in VD. However, extensive research is imperative to pinpoint the precise mechanism and efficacy.

## 4 Potential role of mesenchymal stem cell extracellular vesicles in VD diagnosis

The diagnosis of VD usually involves multiple aspects, including clinical assessments, cognitive evaluations, biomarker analyses, imaging, and laboratory testing (Frantellizzi et al., 2020). Nonetheless, the diagnostic criteria for VD lack uniformity, and varied medical entities or researchers might employ distinct diagnostic criteria, which may lead to differences in outcomes. Concurrently, these diagnostic techniques come with inherent challenges and constraints. They could be swayed by subjectivity, practitioner expertise, and the specificity and sensitivity of biomarkers might be restricted. Occasionally, they might be confounded with other ailments, such as AD (Maclin et al., 2019). Intracranial ultrasound, a non-invasive, replicable, and inexpensive and cost-effective method (Siniscalchi et al., 2021) is widely adopted in current clinical practice. However, it still has challenges such as being limited by the skull and intracranial anatomy and not being able to fully observe deep arteries. As such, there is an imperative to identify a low-risk, high-accuracy, non-invasive biomarker to compensate for the prevailing inadequacies in VD detection methodologies.

There is a growing interest in EVs as potential diagnostic agents reflecting disease states (Joo et al., 2023). MSC-EVs carry a plethora of bioactive molecules, which may serve as potential biomarkers for early diagnosis and disease monitoring. The expression of exosomal miRNA-223-3p is elevated in the plasma of patients with CSVD, especially at the onset of cognitive impairment (Zhao et al., 2021). Consequently, EVs miRNA-223-3p could be a potential biomarker for CSVD. Additionally, Ma et al. (2022) discovered that in early VD mice, levels of MSC-EVs and miR-132-3p were significantly diminished. Re-injecting miR-132-3p-rich MSC-EX restored synaptic and cognitive functions, suggesting miR-132-3p levels could serve as an early diagnostic indicator (Ma et al., 2022). EVs miR-154-5p levels are elevated in VD patients (Han et al., 2022). A decrease in miR-154-5p results in reduced ROS and increased superoxide dismutase in endothelial progenitor cells, indicating that EVs miR-154-5p might be a viable biomarker and therapeutic target (Han et al., 2022). Changes in miRNA expression in CSF or peripheral blood EVs might reflect the onset and progression of VD. Using TaqMan low-density array and single TaqMan assays, miR-10b*, miR29a-3p, and miR-130b-3p were identified and confirmed to be significantly downregulated in VD patients compared with unaffected controls (NC), and the receiver operating characteristic curve showed that the reduction in its levels can also distinguish patients with VD and AD from those with NC (Ragusa et al., 2016). As acquiring CSF EVs is highly traumatic nature of CSF exosome acquisition, the selection of peripheral blood EVs miRNAs as specific markers is more advantageous than the selection of CSF EVs miRNAs (Cui et al., 2021). EVs miRNAs present a more favorable option; although EVs miRNAs offer diagnostic potential, large-scale clinical studies are essential to ascertain their utility in conditions such as VD. Furthermore, it is crucial to examine whether peripheral blood EVs miRNA levels are influenced by factors such as gender, race, inflammatory agents, lifestyle, and age (Cui et al., 2021). This could be further validated by integrating cognitive and imaging data.

Moreover, MSC-EVs contain a range of neuroprotective and vascular protective molecules, including GDF-15 (Xiong et al., 2021), VEGF (Olejarz et al., 2020), and TGF-β. Changes in these molecules may occur early in VD and could relate to neuronal and synaptic dysfunction, as well as vascular damage. As mentioned previously, VD often entails an increased inflammatory response that might exacerbate VD symptoms. MSC-EVs can mitigate this inflammatory response by delivering anti-inflammatory molecules. Monitoring changes in molecules such as NEP, IDE, IL-10, and TGF-β (Ding et al., 2018), or the neuroprotective factor AMPK (García-Prieto et al., 2015), might assist in assessing the inflammation’s severity and progression, supporting diagnostic efforts.

It has been demonstrated that MSC-EVs can serve as clinical diagnostic markers in other domains. Exosome counts are notably higher in cancer patients compared to healthy people (Suchorska and Lach, 2016). As a result, EVs RNA has been extensively employed in diagnostic and prognostic studies of various cancers (Zhu et al., 2019). The reliability of MSC-EVs as clinical diagnostic markers in other areas bolsters the plausibility of their use in VD diagnosis.

In summary, the potential utility of MSC-EVs as biomarkers in VD diagnosis is highly anticipated. Monitoring bioactive molecule changes within MSC-EVs could furnish valuable insights for early VD diagnosis, disease monitoring, and therapeutic effect evaluation. However, more research is needed to validate these potential biomarkers and ascertain their clinical feasibility and accuracy.

## 5 Prospects of MSC-EVs in the treatment of VD

### 5.1 Feasibility of MSC-EVs in the treatment of VD

MSC-EVs have emerged as a preferred treatment for various diseases, presenting a safe and effective alternative to stem-cell-free therapy (Wang Z. G. et al., 2020). MSC-EVs therapies are simpler to administer and safer than whole-cell-based therapies (Keshtkar et al., 2018). EVs are more stable and modifiable than MSCs and pose no tumor formation risk. Given their nano-size and lipid bilayer structure, EVs can readily traverse biological barriers to reach target organs (Ma et al., 2020). It is noteworthy that the composition of MSC-EVs can be manipulated by pre-treating MSCs *in vitro* to produce disease-specific, MSC-based immunosuppressive products. This innovative strategy could introduce a novel cell-free therapeutic approach for autoimmune and inflammatory diseases (Harrell et al., 2019a). Because of their compact size, minimal immunogenicity, extended half-life, neuroprotective effects (Yari et al., 2022), and ease of procurement, MSC-EVs are emerging as prominent delivery vectors (Vizoso et al., 2019). Various preclinical studies have revealed the preventive or therapeutic potential of MSC-EVs in diverse disease animal models (Yaghoubi et al., 2019). Specifically, MSC-EVs have demonstrated therapeutic roles in colorectal, liver injury, pulmonary inflammation, kidney, autoimmune and inflammatory eye diseases, and heart diseases (Harrell et al., 2019a). They have also shown efficacy in neurodegenerative disease rat models, including reducing pathology and ameliorating cognitive dysfunction in VD (Ma et al., 2022). Thus, MSC-EVs present a promising therapeutic avenue for VD.

MSC-EVs can restore neuronal memory and synaptic plasticity by releasing small vesicles containing bioactive molecules (Chen et al., 2021). They promote the proliferation, migration (Olejarz et al., 2020), and tube formation of endothelial cells, activate angiogenesis-related signaling pathways, and release angiogenic regulators. This facilitates the angiogenesis process, improves cognitive dysfunction, and reduces the symptoms of VD.

Furthermore, the widely used antioxidant catalase cannot be delivered to the brain due to the BBB. MSC-EVs, capable of being specifically modified, can traverse the BBB and deliver therapeutic molecules (antioxidant catalase) to damaged regions reaching previously inaccessible areas and evade immune system to avoid clearance once inside the membrane layer (Xia et al., 2021). After crossing the BBB, MSC-EVs mitigate oxidative stress using antioxidant enzymes and molecules. They also enhance the expression of Nrf2 and HO-1, boosting cellular antioxidant capacity, and ameliorating cognitive impairment (Liu et al., 2022).

### 5.2 Advantages and challenges of EVs in VD therapy

MSC-EVs have been extensively researched in various drug delivery studies because of their immune properties, tumor-homing abilities, and elastic properties (Sun et al., 2021). Their natural origin allows them to function well *in vivo*. As cell-free entities, MSC-EVs address the myriad safety concerns associated with long-term viable MSC transplantation. Such concerns encompass uncontrolled cell differentiation, malignant transformations, and rejection due to allogeneic immune response activation from MHC-mismatched receptors (Harrell et al., 2021). The BBB is an important barrier for drug delivery to the brain, thus prevents most substances from entering the brain from the bloodstream, allowing only minute molecules can cross the BBB (Teleanu et al., 2022) to precisely control the brain microenvironment and neural activity (Han, 2021). It severely hinders the delivery of drugs to the brain and the efficiency of various systemic therapies for brain disorders (Han, 2021). MSC-EVs, capable of being specifically modified, can deliver therapeutic molecules to damaged regions and traverse the BBB, reaching previously inaccessible areas (Xia et al., 2021). For instance, EVs -associated miR-105 can downregulate the expression of zonula occludens-1 (ZO-1), a fundamental component of tight junctions. This impairs endothelial barrier function (Zhou et al., 2014), enabling MSC-EVs to deliver therapeutic molecules precisely. However, despite these promising experimental results, substantial research is still required to universalize MSC-EVs as a treatment for VD for a broader population. The production and purification techniques of MSC-EVs demand further refinement, given their intricate nature and the specialized laboratory techniques, which may limit the feasibility of large-scale production and clinical application (Hettich et al., 2020). Challenges exist in targeting MSC-EVs, drug delivery efficiency, and delivery mode (Harrell et al., 2019b). Although intravenous (IV) injection is the most common route of delivery (Wiklander et al., 2015), EVs in the bloodstream are easily cleared by macrophages, resulting in a short half-life (from minutes to hours) (Xu et al., 2021). In addition, EVs administered through this pathway have off-target effects, and they are more likely to reach the kidneys, liver, and spleen when administered. *In vivo* neuroimaging studies have shown that nasal administration of EVs derived from MSCs allows them to cross the BBB more efficiently than IV administration. Intranasal administration of MSC-EVs has been evaluated as a noninvasive and effective route to the brain (Long et al., 2017). Comparative studies are needed to analyze the functional efficiency of various administration routes within the same model (Guy and Offen, 2020). In addition, some studies used only one dose, and it is necessary to explore the possible dose-dependent protective effects of MSC-ev and MSC-EVs (Xia et al., 2019; Sun et al., 2021; Hedayat et al., 2023) Additionally, variations in the isolation and application processes in different studies could lead to diverse effects from identical regulatory proteins and miRNAs. For consistent outcomes from MSC-EVs and MSC-EVs, further exploration and research are required (Guillen et al., 2021). In summary, while MSC-EVs hold promise for treating VD, many scientific, technical, and clinical challenges remain before they become a standard clinical practice. With continuous scientific advancement and deepened research, these hurdles are expected to be cleared, leading to more effective treatment options for VD patients.

### 5.3 Clinical experiments of MSC-EVs in VD

A recent search on ClinicalTrials.gov (as of October 19, 2023) with the keyword “stem cell EVs” revealed 39 studies. However, no study was found when “vascular dementia” was included in the “condition or disease” search field. Nevertheless, our article has analyzed and demonstrated the potential of stem cell-derived EVs in treating VD. Given the promising results from clinical trials in other diseases, such as gastrointestinal fistulas (Nazari et al., 2022), COVID-19 (Zhu et al., 2022), we believe that clinical trials for VD using mesenchymal stem-cell-derived EVs may yield optimistic outcomes.

## 6 Future research directions and challenges

In recent years, MSC-EVs therapy has become a novel treatment alternative for numerous diseases (Mendt et al., 2018). Globally, the number of companies interested in MSC-EVs as a therapeutic tool has reached 134 year by year (Lotfy et al., 2023). EVs from MSCs show promising potential in diagnosing and treating VD. However, the functional mechanisms of EVs are not yet fully understood. Further research is imperative to exploit the therapeutic role of MSC-EVs (Keshtkar et al., 2018). Moreover, standardized protocols for the isolation of EVs should be developed to increase their purity while maintaining biologically active cargo contents. Although the quadrangular proteins CD9, CD63, and CD81 are often used as biomarkers for exosome isolation, they cannot be reliably used for immunoaffinity isolation of EVs because the expression of these proteins varies widely across different cell types (Torres Crigna et al., 2021). Therefore, a common biomarker for EVs isolated from different cell types, including stem cells, needs to be identified in future studies. Concurrently, it is crucial to delve into the large-scale cultivation and isolation technology of MSCs, refine methods for the long-term preservation of EVs, the rapid and accurate determination of EVs concentration, the quality control of EVs, purification, and transplantation conditions (Tang et al., 2021) and establish standards for the characterization and drug delivery (Keshtkar et al., 2018). Despite its potential, the clinical application of MSC-EVs remains limited. Key parameters need addressing for the translation of MSC-EVs therapy from preclinical studies to the clinic needs to address key parameters (Gimona et al., 2017). With the rapid advancements in bioengineering and cell modification techniques, future endeavors in the exosome domain might focus on engineering or modifying the exosome surface and content, enhancing specificity for applications in complex medical fields (Mendt et al., 2019). While challenges persist, the prospects of EVs in both fundamental research and clinical applications are exciting and warrant further exploration.

## 7 Conclusion

MSC-EVs are increasingly utilized in clinical studies for various diseases and are viewed as a promising cell-free therapeutic avenue. Numerous clinical trials have reported their safety and potential efficacy. In conclusion, this research explored the possible application of MSC-derived EVs in the diagnosis and treatment of VD. It highlighted the involvement of MSC-EVs in the pathophysiology of VD and the potential diagnostic role of EVs in VD, positioning them as an emerging therapeutic strategy. Current challenges and future research avenues were also discussed. Through interdisciplinary collaborations and relentless research efforts, we aspire to offer more efficient treatment methods for VD patients, enhancing their quality of life.

## Author contributions

ZL: Writing – original draft, Investigation, Software, Visualization, Writing – review & editing. LC: Funding acquisition, Writing – original draft, Writing – review & editing. LZ: Supervision, Writing – review & editing. CS: Data curation, Investigation, Writing – review & editing. SW: Data curation, Formal analysis, Writing – review & editing. LW: Formal analysis, Funding acquisition, Writing – review & editing. YQ: Data curation, Formal analysis, Writing – review & editing. CL: Writing – review & editing. YX: Funding acquisition, Writing – review & editing. XZ: Funding acquisition, Writing – review & editing.
